# Natural Polyhydroxyalkanoates—An Overview of Bacterial Production Methods

**DOI:** 10.3390/molecules29102293

**Published:** 2024-05-13

**Authors:** Ivo Fukala, Igor Kučera

**Affiliations:** Department of Biochemistry, Faculty of Science, Masaryk University, Kotlářská 267/2, CZ-61137 Brno, Czech Republic; ivo.fukala@mail.muni.cz

**Keywords:** PHA, PHB, autotrophy, methylotrophy, heterotrophy

## Abstract

Polyhydroxyalkanoates (PHAs) are intracellular biopolymers that microorganisms use for energy and carbon storage. They are mechanically similar to petrochemical plastics when chemically extracted, but are completely biodegradable. While they have potential as a replacement for petrochemical plastics, their high production cost using traditional carbon sources remains a significant challenge. One potential solution is to modify heterotrophic PHA-producing strains to utilize alternative carbon sources. An alternative approach is to utilize methylotrophic or autotrophic strains. This article provides an overview of bacterial strains employed for PHA production, with a particular focus on those exhibiting the highest PHA content in dry cell mass. The strains are organized according to their carbon source utilization, encompassing autotrophy (utilizing CO_2_, CO) and methylotrophy (utilizing reduced single-carbon substrates) to heterotrophy (utilizing more traditional and alternative substrates).

## 1. Introduction

**Figure 1:**
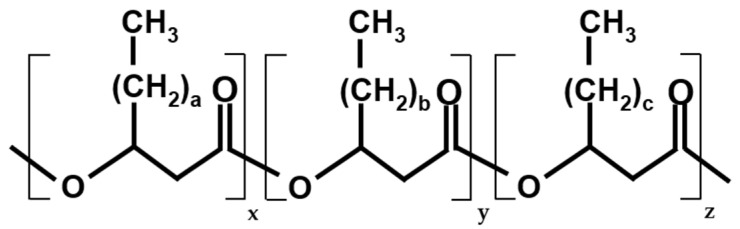
General structure of PHA (co-)polymers. a,b,c—number of additional methylene bridges in the chain (0–14), x,y,z—number of monomer units of each hydroxyalkanoate in the polymer chain (0 → ∞).

The term ‘bio-based polymers’, recommended by IUPAC and colloquially referred to as ‘bioplastics’, refers to polymeric substances produced from renewable resources and at least partially of biological origin. This includes compounds whose starting material was produced naturally, such as starch, cellulose, and lactic acid, but which was later re-molded or polymerized abiotically. Such material is typically hydrophilic and brittle. There are also more robust materials, such as polylactic acid, but it has been found that these materials accumulate in the environment similarly to petrochemical plastics [1]. Bio-based polymers can also refer to compounds that are produced entirely through biological means. These substances are known as polyhydroxyalkanoates (PHAs), which serve as carbon and energy reserves for cells. PHAs are biodegradable in the environment due to their biological origin, while still retaining some characteristics of petrochemical plastics. These polymers are predominantly employed in the field of packaging [2,3], with ongoing research exploring their potential applications in the medical field [4] and in agriculture [5]. Figure 1 illustrates the general structure of PHAs.

PHAs are divided into two categories based on the length of the monomer used for polymerization: short-chain-length PHAs (scl-PHAs), which use C3–C5 substrates, and medium-chain-length PHAs (mcl-PHAs), which use C6+ substrates (usually up to C14). The most widely produced storage polymer is scl-PHA, specifically the C4-derived poly(3-hydroxybutyrate) (PHB). It can form copolymers with C5-derived polyhydroxyvalerate (PHV), which improves the mechanical properties of the resulting material. C3- and C4-derived polyhydroxypropionate and poly(4-hydroxybutyrate) are not naturally occurring storage polymers. However, their desirable mechanical properties have led to efforts to produce these copolymers in bulk through genetic modifications to organisms or using carbon precursors. While scl-PHAs have been studied more extensively, mcl-PHAs such as polyhydroxyhexanoate (PHH) have higher elasticity and a lower melting point. Therefore, copolymers of scl- and mcl-PHAs are desirable due to their decreased brittleness. Table 1 compares the characteristics of various PHA copolymers.

**Figure 2:**
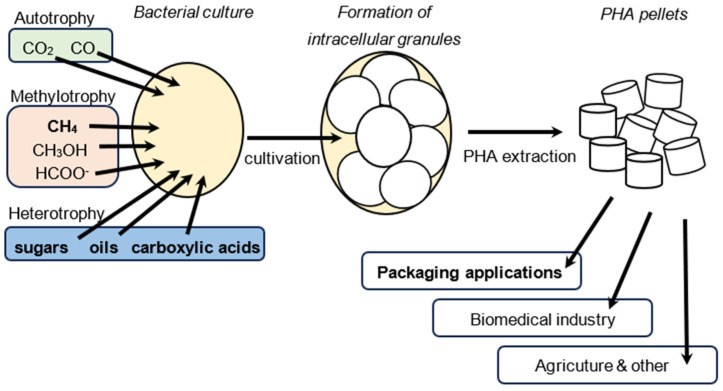
Diagram of PHA production by microorganisms. The carbon substrate is assimilated by the bacterial strain, resulting in the formation of intracellular PHA granules. These serve as carbon and energy storage for the cell. The PHA granules can be extracted and processed into pellets or powder, which can then be further processed to be used, for instance, as a packaging material.

**Figure A1:**
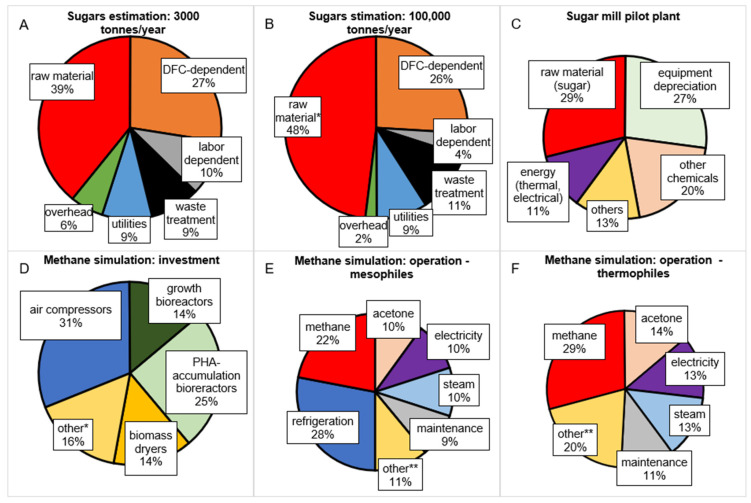
Visualization of distribution of PHA production costs using three studies. (**A**,**B**) Estimation. Carbon source accounts for 70–80% of total raw material costs. DFC—direct fixed capital (initial investment costs) [13], (**C**) pilot plant [14], (**D**–**F**) simulation [15]. *—other represents centrifuges, coolers, filtration systems, etc. **—other represents supervision, labor, administration, solids handling, etc.

PHA production is highly dependent on the organism, cultivation, and extraction method used. *Cupriavidus necator* (previously called *Ralstonia eutropha*) is one of the most studied organisms for PHA production due to its ability to naturally produce PHAs in high amounts and due its high metabolic versatility. PHA accumulation can also be increased by a high C/N ratio, depletion of N, S, and P, or a low rate of respiration (resulting in more reducing conditions in the cell). The primary disadvantage of PHAs is their high production cost, which is mainly due to the expensive carbon source that can account for up to 40% of total costs. A study conducted in 1997 estimated the cost of investments and maintenance, using sugars as a carbon source, and the extraction of PHAs using hypochlorite digestion and a detergent [13]. In 1995, the Copersugar company constructed a pilot plant in a sugar mill in Brazil to produce PHB. The plant processed biomass with a PHA content of 65–70%, which was extracted using non-halogenated solvents (medium-length alcohols) [14]. A 2016 study used a computer simulation to evaluate the potential for PHB production from methane, with extraction being carried out using acetone. The use of thermophilic organisms would eliminate the need for cooling, which is the most expensive operation [15]. For an overview of the cost distribution of PHA production, refer to Figure A1 in Appendix A. The current commercial producers of PHA include, among others, Danimer Scientific (Bainbridge, GA, USA), Shenzhen Ecomann Biotechnology Co., Ltd. (Shenzhen, China), Kaneka Corporation (Osaka, Japan), RWDC Industries (Singapore), TianAn Biologic Materials Co., Ltd. (Ningbo, China), Newlight Technologies LLC (Huntington Beach, CA, USA), and Biomer (Schwalbach, Germany). These producers primarily utilize sugars and vegetable oils as raw materials. Figure 2 provides an overview of the production of PHAs using microorganisms.

In the environment, polyhydroxyalkanoates (PHAs) are degraded by microbial PHA-degraders that excrete PHA depolymerases, which themselves are not necessarily PHA producers. These include bacteria and fungi. Some bacterial lipases are also capable of degrading PHAs [16]. Abiotic factors, such as elevated temperature, low pH, UV radiation, high relative humidity, and the surface area of the polymer (plates vs. granules), facilitate PHA degradation. A review of various studies on the environmental degradation of PHA was conducted [17]. The rate of degradation is also influenced by the diversity of the microbial community. The time frame for degradation varies between weeks and months. The majority of studies indicate that copolymers, such as PHBV, degrade at a faster rate than PHB due to their lower crystallinity and more porous surface [17]. A more recent study has highlighted the significance of relative humidity in soil. Samples were observed to degrade within two weeks at 100% relative humidity, whereas degradation was negligible within the same time frame at 40% humidity [18].

## 2. Metabolism of PHAs

### 2.1. Biosynthesis

**Figure 3:**
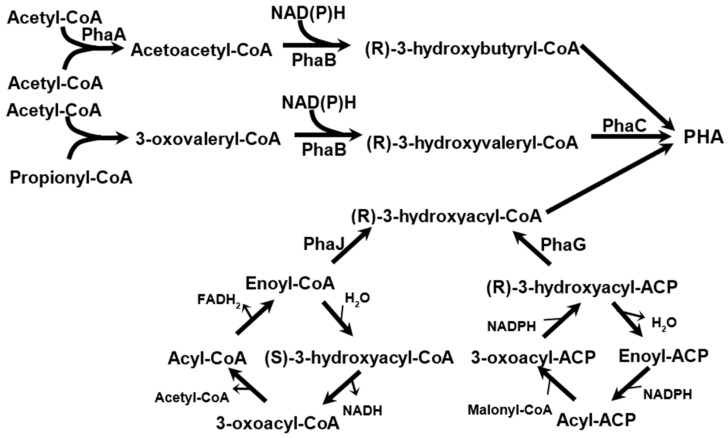
Pathways to form scl- and mcl-PHAs.

Figure 3 shows general pathways related to the synthesis of storage PHA. These PHAs can be produced by bacteria and archaea that inhabit soil and water environments.

The main pathway for PHB synthesis begins with the condensation of two molecules of acetyl-CoA into acetoacetyl-CoA, which is then reduced to 3-hydroxybutyryl-CoA, the active form of 3-hydroxybutyrate. These activated monomers are then polymerized into PHB. The enzymes that catalyze these reactions are commonly referred to as PhaA, PhaB, and PhaC. Other acyl-CoA compounds, such as valeryl-CoA, can be utilized for polymerization. Valeryl-CoA can be synthesized from acetyl-CoA and propionyl-CoA. The synthesis of mcl-PHAs involves the use of (R)-3-hydroxyacyl-CoAs, which can be obtained through the beta-oxidation of fatty acids (using enzyme PhaJ [19]) or during the de novo synthesis of fatty acids (using enzyme PhaG [20]).

### 2.2. PHA Granules

Synthetized PHA chains are known to form intracellular inclusion bodies, which are commonly referred to as PHA granules. These granules contain amorphous PHA. Subsequent industrial processing results in the denaturation and transformation of these PHAs into a more crystalline structure. The majority of the surface area of a native PHA granule is covered with an amphiphilic protein called phasin (PhaP), which forms a boundary between the hydrophobic PHA chains and hydrophilic cytosol. The expression of the phasin gene is controlled by the PhaR regulator protein, which competes with phasin on the granule surface. Phasin has a higher affinity for the granule and eventually replaces all PhaR, which then binds upstream of *phaP* and represses its expression [21]. The remaining surface of the granule is covered with PHA polymerases and depolymerases. In *C. necator*, an additional protein PhaM anchors the granule to the nucleoid and affects the total number of granules in the cell [22].

There are three main models for granule formation. The first is the micelle model, where PHA polymerases surround a micelle that accumulates synthetized PHA chains. The second is the budding model, in which PHA synthases are located on the cytosolic side of the cell membrane, PHA accumulates in the phospholipid bilayer, and eventually buds into cytosolic side. Finally, the scaffolding model is similar to the micelle model, but nucleation centers are referred to as ‘mediation elements’, which are dark-stained structures that appear during electron microscopy but have not been identified further. A modified micelle model emerged later, where nascent PHA-chains first create a complex with phasins to maintain solubility and later accumulate into larger granules (as reviewed in [23]).

### 2.3. Mobilization of PHAs

The utilization of these storage compounds requires the enzymatic degradation of PHA by PHA depolymerases (PhaZ). This enzyme cleaves the long chain into both monomers of 3-hydroxybutyrate and into oligomers of 3-hydroxybutyrate. The oligomers of 3-hydroxybutyrate require a dedicated enzyme to be further split into monomers [24]. Subsequently, monomeric 3-hydroxybutyrate is oxidized to acetoacetate. After its activation to acetoacetyl-CoA, it can re-enter metabolism. The degradation of mcl-PHA yields fatty acids that can enter beta-oxidation. 

### 2.4. Role of PHAs in Cell Physiology 

Long-chained PHAs are well-known for their role as storage compounds in bacteria and archaea. Nevertheless, short methyl-esterified dimers and trimers of 3-hydroxybutyrate are highly effective antioxidants against hydroxyl radicals (3-fold more effective than glutathione and 11-fold more effective than ascorbic acid and monomeric 3-hydroxybutyrate) in cells that possess PhaC and PhaZ enzymes. These findings provide new insights into the utilization of PHA reserves in microorganisms that inhabit stressful environments and in endophytic microorganisms that infect plant cells [25].

Small amounts of PHB seem to be present in all organisms. This has been evidenced by the detection of PHB in samples from organisms that do not form storage PHA granules, including *E. coli*, yeast, spinach leaves, and animal and human tissues. The elevated concentration of PHB in competent *E. coli* cells suggests its importance for exogenous DNA uptake during bacterial transformation [26]. Furthermore, it has been identified in human plasma [27]. Oligomers of PHB (100–200 units) have been observed to create a complex with polyphosphates and Ca^2+^ ions to form channels that facilitate ion transport, as reported in the membranes of *E. coli* [28]. Furthermore, PHB has been implicated in the formation of the mitochondrial permeability transition pore during apoptosis, as reported for tissue samples from rat livers [29]. Additionally, short PHB chains were found to be covalently linked to the outer membrane protein OmpW of *E. coli*. This may be involved in its targeting to the outer membrane and assembly [30]. Furthermore, data indicate that histones and analogous DNA-binding proteins in prokaryotes are covalently modified by oligomeric PHB [31].

The existence of oligomeric PHB in organisms that lack PhaC has remained a mystery, as it has been unclear how these polymers can be synthesized. In *E. coli*, it has been demonstrated that YdcS, a component of a putative periplasmic ABC transporter, exhibits PHB synthase activity [32]. A simple database search reveals that the sequence of this protein is widely distributed among bacteria. To date, no eukaryotic protein with a similar function has been reported. The significance of non-storage PHB for cellular physiology remained obscure for a long time, due to the lability of its connection and the polymer’s absence in protein crystallographic studies, which is attributed to sample preparation methods that do not preserve it [33].

## 3. Determination of PHA Content in Cells

There are a number of different methods that can be employed to determine the content of polyhydroxyalkanoates (PHA) in biomass. The intracellular PHA granules can be observed in high definition using transmission electron microscopy (TEM), although this method requires laborious sample preparation and expensive equipment. A rapid and relatively simple approach is fluorescence microscopy using Nile Red dye, which binds to intracellular granules (emission maximum around 610 nm) [34]. However, due to its lipophilic nature, this dye is not suitable for quantitative analysis.

The content of PHA can be determined gravimetrically by its extraction into organic solvents, which are subsequently evaporated or dissolved PHAs are selectively precipitated. Nevertheless, such extractions do not ensure sufficient PHA purity. Indeed, the purity of the extracted PHA can vary considerably, ranging from 66 to 96%, depending on the pretreatment of the cells [35]. PHB can also be dehydrated to crotonic acid, which can be determined via high-performance liquid chromatography (HPLC) with a UV detector unit. A traditional method for determining the composition of PHA is transesterification, which converts the polymer into volatile methylesters. These can be identified by gas chromatography. In recent years, Fourier transform infrared (FTIR) spectroscopy has emerged as a valuable tool for the qualitative and quantitative determination of PHAs [36,37]. This method can be applied to intact cells, eliminating the need for laborious pretreatment. For further details on the methodologies employed for the determination of PHA, please refer to [38].

## 4. Extraction Methods

It may be useful to provide a brief summary of the methods for subsequent PHA extraction from biomass. To date, the most effective method for disruptive extraction in terms of purity, recovery rate, and high levels of polymerization of extracted PHAs (high molecular weight) is the use of halogenated organic solvents, such as chloroform. Their high ecotoxicity raises concerns about the environmental impact and limits their use in bulk. In an effort to reduce their use, researchers have explored the possibility of pretreating the cells with supercritical CO_2_ [39], or replacing these solvents entirely with non-halogenated solvents, such as alcohols [14], butyl acetate [40], or methyl isobutyl ketone [41]. A combination of sonication and detergents [42,43], sonication and extraction by NH_3_ [44,45], or the use of subcritical water [46] have also been attempted, with varying levels of success. Additionally, cells were genetically modified to excrete PHAs, as observed in the case of *E. coli*, which excreted 36% PHB [47]. A mutant strain of *Alcanivorax borkumensis* was observed to excrete PHA in large but undetermined amounts [48].

## 5. Overview of Artificial Modifications to Enhance PHA Production

**Figure 4:**
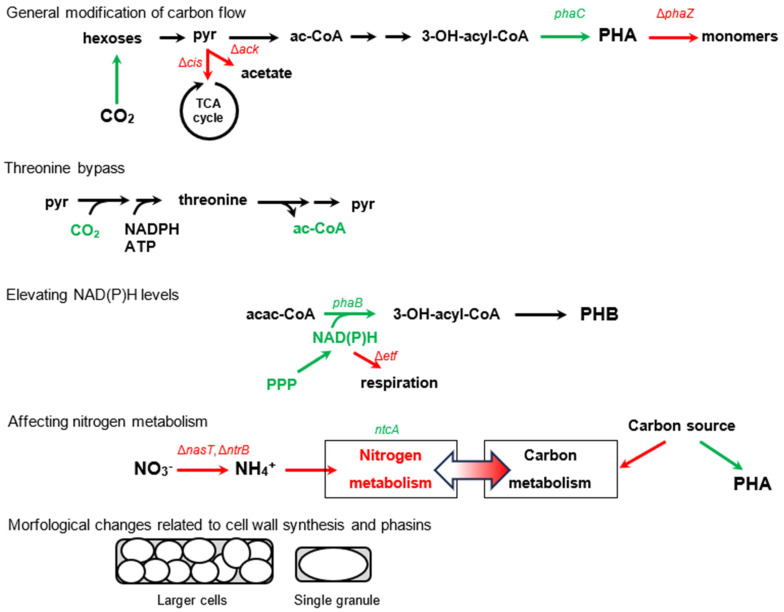

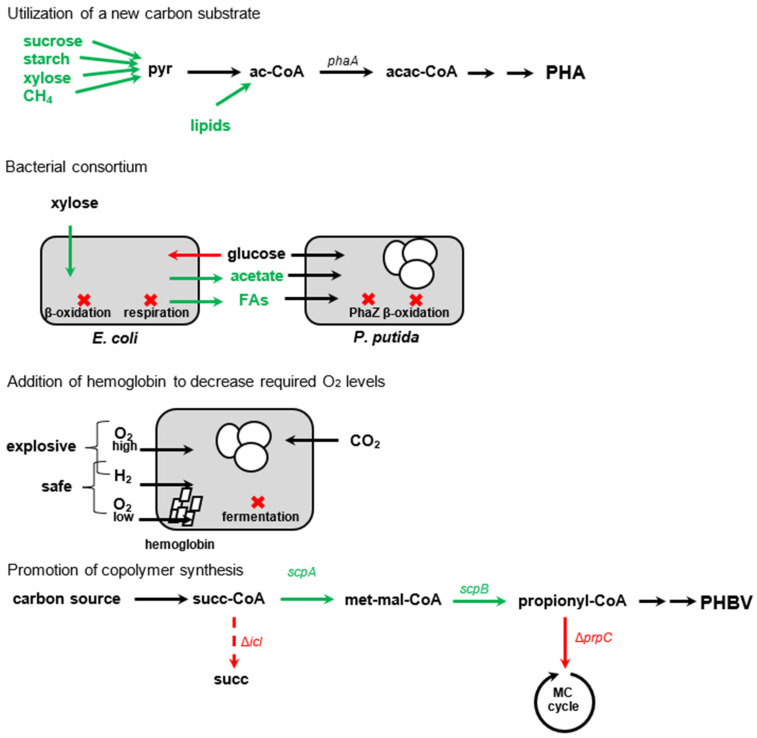
Overview of some modifications of metabolic pathways to promote PHA accumulation. Newly introduced or stimulated paths are in green; suppressed paths are in red. Red crosses indicate paths that have been suppressed in cells (grey rounded rectangles). Abbreviations: pyr—pyruvate, ac−CoA—acetyl coenzyme A, cis—citrate synthase, ack—acetate kinase, TCA—tricarboxylic, acac−CoA—acetoacetyl coenzyme A, PPP—penthose phosphate pathway, etf—electron transfer flavoprotein, FAs—fatty acids, succ−CoA—succinyl coenzyme A, succ—succinate, met−malo−CoA—methylmalonyl coenzyme A, MC—methylcitrate, scpA—methylmalonyl coenzyme A mutase, scpB—mehylmalonyl coenzyme A decarboxylase, icl—isocitrate lyase, prpC—2−methylcitrate synthase.

Recently, modifications to enhance PHA production have been reviewed [49]. Figure 4 provides an overview of several modifications that have been employed to enhance PHA accumulation, which will be discussed in greater detail in this section.

One strategy involves modifying the carbon flow towards PHA accumulation by overexpressing genes related to PHA synthesis (*phaC*) [50] (from 6% PHA content to 8% PHA content), carbon assimilation (Calvin–Benson cycle) (from 49% to 58%) [51], or decreasing catabolism by downregulating citrate synthase within the citric acid cycle (from 49% to 55%) [52]. To utilize the excess reducing power, a “threonine bypass” was developed to assimilate CO_2_ during growth on glucose, which was further enhanced by the addition of stress-tolerant genes (from 34% to 57%) [53,54].

To reduce acetoacetyl-CoA and promote the synthesis of PHA precursors, one can increase the concentration of NAD(P)H by overexpressing pentose phosphate pathway enzymes (co-expression with *phaBRC* for precursors, 2.6-fold increase in g/L titre, 24% PHA max) [55] or by deleting electron transfer flavoproteins (from 84% to 90%) [56]. As carbon and nitrogen metabolisms are interconnected, modifying nitrogen regulators can redirect carbon flow towards PHAs. For example, deleting sensors of NH_4_^+^ (NtrB) (from 17% to 93 93%) [57] or nitrate (NasT) (from below 1% to 15%) [58], or by overexpressing the sensor related to 2-oxoglutarate concentration (NtcA) (from 2% to 5%) [59], can be effective.

The amount of PHA content in cells is limited by the size of the cell wall. When specific acyltransferases are deleted, the compactness of the outer cell membrane decreases, resulting in an increase in PHA content (from 70% to 81%) [60]. Knocking out certain phasin genes and overexpressing genes for cell-fission-ring-blocking proteins can cause PHA to form a single large granule, which increases in size along with the cells [61]. Although larger granules could be beneficial for subsequent PHA extractions, the percentage of PHA was somewhat lower in comparison to single-granule non-producing strains (drop from 80% to 73%). 

There have been many modifications made to strains in order to increase their metabolic versatility. For instance, transport systems and kinases have been added for glycerol (cultivation time dropped to 72 h from 268 h, and PHA content increased from 60% to 64%) [62], sucrose (resulting in 74%) [63], xylose (up to in 73%, together with NDH-II knockout) [64], and raw starch (up to 63%) [65]. The addition of lipases has also been reported (up to 66%) [66]. Lignocellulose can serve as a carbon source through the introduction of PHA synthesis into natural lignocellulose utilizers, which are resistant to various toxins in the substrate (39%, mixture of xylose and glucose originating from wheat straw) [67]. It can also be utilized by modifying strains capable of growing on both lignocellulose and methane (1%) [68]. A bacterial consortium was created in which xylose is first converted to acetate by *E. coli* before being utilized for PHA production by *Pseudomonas putida*. This was achieved through the deletion of glucose uptake, suppression of respiration, promotion of fatty acid export, deletion of PHA depolymerase, and beta-oxidation pathway (1.30 g/L, %PHA not determined) [69]. To ensure safety, aerobic cultivation on H_2_ requires a low concentration of O_2_. In this instance, PHA accumulation was increased by deleting enzymes related to fermentation, such as lactate dehydrogenase, acetate kinase, and isocitrate lyase, and by introducing low-O_2_-tolerant hemoglobin to improve O_2_ diffusion in the cell (from 39% to 50%) [70]. Modifications can also be used to produce copolymers by increasing the accumulation of propionyl-CoA, which can be achieved by deleting isocitrate lyase, succinate dehydrogenase, and methylcitrate synthase (65% with 25% PHV) [71].

## 6. Application of PHA-Producing Strains

In this context, PHA-producing organisms are classified into groups based on their carbon source, source of electrons, and source of energy to power metabolism. Organisms can obtain carbon in two primary forms: as oxidized inorganic compounds, such as CO_2_ and CO, which are utilized in autotrophic processes, or in its reduced organic form, which is employed in heterotrophic processes. By convention, an autotroph must obtain at least 50% of its carbon from oxidized carbon forms and vice versa [72]. On occasion, the capability to metabolize one-carbon (1C) organic compounds, such as methanol and formate, is designated as “methylotrophy” to differentiate it from growth on two-carbon (2C+) substrates [73]. This distinction is maintained here. In order to reduce oxidized carbon and/or use it for ATP production via oxidative phosphorylation, a source of electrons is necessary. This donor can be either an organic (organotrophy) or inorganic (lithotrophy) compound. The energy for the system can come from conversion of chemical bonds (chemotrophy) towards a state with minimum energy, or from electromagnetic radiation (phototrophy). To illustrate this with a more specific example, there exist cyanobacteria that produce PHA and use carboxylic acids as a growth source. However, they acquire energy from sunlight, making them photo-organoheterotrophs. In contrast, more traditional PHA producers, which grow on sources such as glucose, obtain energy by oxidizing the carbon substrate, thus classifying them as chemo-organoheterotrophs. PHA-producing cyanobacteria that fix CO_2_ via oxygenic (O_2_ evolving from inorganic H_2_O) photosynthesis are photolithoautotrophs. Table 2 provides a summary of possible combinations. 

The main parameters related to cell cultivation for PHA accumulation are the weight of PHAs per dry mass of cells (*w*/*w*) and the titer of PHA in the volume of cell suspension (g/L). While a high PHA yield in grams per liter is desirable, the concentration of cells in the suspension can be increased by filtration or centrifugation. The *w*/*w* ratio determines the amount of the carbon source that is converted into PHAs rather than PHA-unrelated biomass. Although a high conversion ratio may not be necessary for inexpensive carbon sources, the ballast biomass must still be separated, which can require expensive and/or ecotoxic chemicals and can negatively impact PHA purity (as discussed in *Extraction of PHAs*). Therefore, we were primarily interested in the strains that produce the highest PHA (*w*/*w*), although we are aware that scaling up such cultivation may pose challenges. This section mainly concerns the production of PHB, which is the most widely studied polymer, with several copolymers and mcl-PHA mentioned. The production of copolymers usually requires the utilization of more direct or indirect precursors, such as propanol, propionic acid, or valeric acid. Table 3 shows an overview of categories subsequently discussed here.

### 6.1. Autotrophy

**Figure 5:**
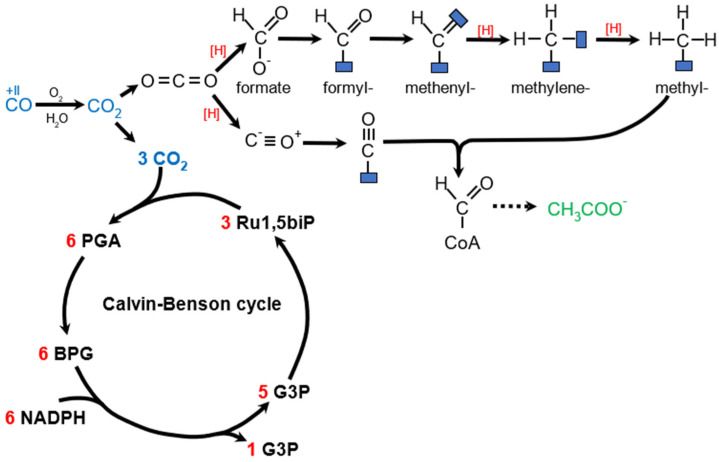
Autotrophic pathways related to PHA production. CO is oxidized to CO_2_ in aerobic or anaerobic conditions. Upper part: schematic representation of Wood–Ljungdahl pathway. For simplicity, all reducing equivalents are represented by [H]. Bottom part: schematic representation of Calvin–Benson cycle. Ru1,5biP—ribulose−1,5−bisphosphate, PGA—3−phosphoglycerate, BPG—1,3−bisphosphoglycerate, G3P—glyceraldehyde−3−phosphate.

The reduction in CO_2_ is accomplished through either the oxidation of inorganic compounds such as H_2_ (chemolithoautotrophy) or the utilization of sunlight to split inorganic compounds and obtain electrons (photolithoautotrophy). The most well-known example of the latter is oxygenic photosynthesis, which utilizes H_2_O as the electron source and fixes CO_2_ using the Calvin–Benson cycle (reductive pentose phosphate pathway), which utilizes the RuBisCO enzyme. In this pathway, CO_2_ reacts with ribulose-1,5-bisphosphate to form glyceraldehyde-3-phosphate. RuBisCO itself is estimated to be the most abundant protein on Earth (0.7 Gt total) [74]. H_2_ can serve as an electron source when oxidized by O_2_ in aerobic hydrogenotrophs utilizing Calvin–Benson cycle, such as *C. necator*. Anaerobic chemolithoautotrophs are non-native PHA producers that utilize the Wood–Ljungdahl pathway (WLP, reductive acetyl-CoA pathway) for both CO_2_ assimilation and energy production. This pathway involves the subsequent reduction of CO_2_ to formate and acetyl-CoA, which is either assimilated or used for ATP production and dissimilated by acetogens. Other autotrophic pathways not associated with PHA production are not discussed here. Figure 5 shows autotrophic pathways related to reported PHA-producing organisms. For more information on autotrophic and methylotrophic strains for PHA production, refer to [75]. 

#### 6.1.1. Photoautotrophy (Sunlight)

Cyanobacteria are a promising source for PHA production due to their oxygenic photosynthesis, which eliminates the need for dangerous chemicals during cultivation. Currently, the main challenge is to suppress the accumulation of glycogen, the preferred storage compound, and increase PHA content. According to a study, a random mutant of *Synechocystis* sp. PCC 6714 was able to increase PHB accumulation by 37% [76]. Similarly, *Synechocystis* sp. PCC 6803, overexpressing RuBisCO genes, produced 39% PHB when they were limited by nitrogen and phosphorus and were supplied with NaHCO_3_ [77]. *Oscillatoria okeni*, on the other hand, is capable of producing 14% PHA with 6% PHV using only CO_2_ during nitrogen limitation. When supplied with 0.4% acetate, PHA increased to 42% with 6.5% PHV [78]. *Anabaena* sp. can produce 40% PHA with 5% PHV through pure autotrophy. The introduction of different concentrations of acetate resulted in an increase in the proportion of PHV, up to 24%, but a decrease in total PHA content, down to 14% [79]. Mixotrophic cultivation with heterotrophic carbon sources can enhance PHA production, as discussed in the *Photoheterotrophy* section.

#### 6.1.2. Hydrogenotrophy (H_2_)

Hydrogenotrophs can utilize H_2_ to power carbon assimilation, either aerobically (using the Calvin–Benson cycle) or possibly anaerobically (using the Wood–Ljungdahl pathway) to produce PHAs. Currently, most H_2_ is produced from fossil fuels, but more sustainable approaches are being considered for the future. All of these methods use H_2_O as a starting material. They include electrolysis (using renewable or nuclear energy), thermochemical production, photocatalytic production, and production by living cells (using cyanobacteria or algae).

The highest reported PHA content was achieved through aerobic cultivation of hydrogenotrophic *C. necator*, which produced 85% PHB a decade ago [80]. H_2_ and O_2_ can form an explosive mixture, so it is necessary to exercise enhanced care. Coupling bioreactors to water electrolysis outputs could improve the economics of such production. For copolymer production, *C. necator* was genetically modified with transgenic PHA synthases and an acyl-carrying protein. This resulted in the synthesis of various scl/mcl-PHA copolymers using only CO_2_ as a carbon source, with a total PHA content of 23–55%. The study found that the highest yields were achieved with 55% PHA and 20% polyhydroxydodecanoate, 46% PHA with 39% polyhydroxydodecanoate, and 42% PHA with 55% polyhydroxyoctanoate (the latter two were obtained by adding acrylic acid to limit beta-oxidation of fatty acids) [81].

Anaerobic hydrogenotrophs, including methanogens and acetogens, are not natural producers of PHAs. However, they could be genetically modified to produce PHAs. The use of anaerobic strains eliminates the need for O_2_ and prevents the formation of explosive mixtures, but there are inherent bioenergetic limitations that must be considered. In order to obtain enough ATP, most of the carbon source must be dissimilated into waste CH_4_ or acetate, rather than being assimilated into PHAs. The net yield of ATP per substrate molecule is only 0.5–2 for methanogens and 0.25 for acetogens [82]. The ratio of carbon assimilation to dissimilation has been experimentally determined for acetogens grown on H_2_: for every 24 carbon atoms, 1 is converted into biomass, 20 into acetate, and 3 are unrecovered during measurements [83]. Therefore, a high intracellular PHA concentration would result in the production of large volumes of side products. However, these side products could be further utilized by employing a mixed culture of PHA-producing hydrogenotrophs together with methanotrophs and/or acetotrophs. It is important to note that chemoheterotrophs require an electron acceptor to obtain energy. Aerated co-culture is not possible because the Wood–Ljungdahl pathway is extremely sensitive to oxygen. However, both methanotrophs and acetotrophs could be denitrifiers or sulfate reducers, requiring either NO_3_^−^ or SO_4_^2−^ in the medium. Alternatively, photoheterotrophs that do not require such oxidant, such as *Rhodobacter capsulatus*, could be used. This bacterium is able to grow anaerobically on acetate as a sole carbon source [84]. The acetogenic bacterium *Clostridium autoethanogenum* has been genetically modified to produce PHAs. However, as its main carbon source is CO, it is discussed in the following section.

#### 6.1.3. Carboxydotrophy (CO)

Carbon monoxide (CO) is naturally produced by volcanic activity, but it is also present in industrial gases, such as syngas, which is made abiotically from various fossil fuels. Syngas can contain up to 60% CO, with the remaining gases mainly consisting of H_2_ and low amounts of CH_4_ and CO_2_. CO is more reduced than CO_2_ and can be used as an electron donor. However, the ability to grow on CO, known as carboxydotrophy, is usually considered a form of autotrophy (e.g., in [85]) because it utilizes oxidized inorganic carbon. To utilize CO, it must first be oxidized to CO_2_ using either O_2_ or H_2_O and can later be assimilated using either the Calvin–Benson cycle or WLP. Although carbon monoxide (CO) is an intermediate of the WLP, it is unlikely that external CO would be utilized directly. This is because carbon monoxide dehydrogenase and acetyl-CoA synthase are connected via a channel that CO travels through [86]. When grown aerobically on 30% CO and limited via sulfur, *Seliberia carboxydohydrogena* Z-1062 was able to accumulate polyhydroxybutyrate (PHB) up to 63% [87]. Some components of crude syngas can inhibit cell growth, but these can be resolved by using charcoal filters [88]. Acetogenic *Clostridrium autoethanogenum* has been genetically modified to produce PHAs using substrates that mimic syngas (50% CO, 20% CO_2_, 20% H_2_, Ar) or steel mill-off gas (50% CO, 20% CO_2_, 2% H_2_, N_2_) [89]. The ratio between carbon in biomass and acetate ranged between 1:8 and 1:13, based on the available data. This is consistent with [83], as it is unclear how much carbon substrate was used to achieve these ratios. A significant amount of CO_2_ was also present, but it is unclear how much of it originated from the oxidation of CO. To enhance PHA synthesis, the authors aimed to minimize the ATP used for cell maintenance. They discovered that a low pH has a negative impact on PHA accumulation. This is because the excreted waste acetate is protonated and flows back into the cell, and its intracellular deprotonation depletes the proton gradient. Increasing the pH [89,90] resulted in a 6% accumulation of PHB [89]. Even with optimized ATP consumption, the ratio of waste to biomass will remain constant, as acetate dissimilation is required for ATP production. Any excess acetate waste must be properly processed or separated.

One potential concern for future PHA production using syngas is its origin from fossil fuels. While biomass production is feasible, the biological conversion of biomass to biogas appears to be a more viable option. It is important to note that, in addition to the danger of forming explosive mixtures with air in case of an accident, CO is highly toxic, unlike H_2_ or CH_4_. Therefore, it requires more a strictly controlled operation.

### 6.2. Methylotrophy

**Figure 6:**
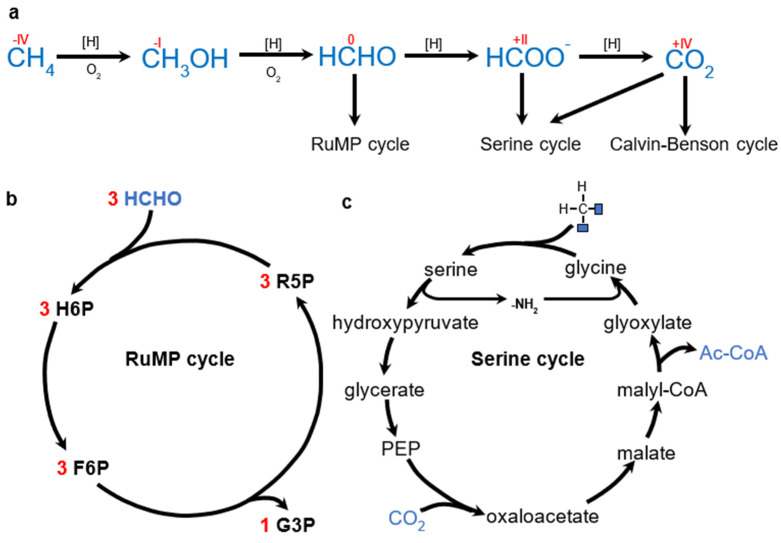
(**a**) Overview of methylotrophic pathways considered for PHA production so far. For simplicity, all reducing equivalents are represented by [H]. (**b**) Schematic representation of RuMP. R5P—ribulose−5−phosphate, H6P—hexulose−6−phosphate, F6P—fructose−6−phosphate, G3P—glyceraldehyde−3−phosphate. (**c**) Schematic representation of serine cycle. Formate is converted to methylene before entering the cycle.

Methylotrophic organisms are able to grow on 1C organic substrates (e.g., CH_4_, methanol, formate). Such reduced 1C substrates require specific assimilation pathways. These include the ribulose monophosphate (RuMP) cycle and the serine cycle (other pathways such as the xylulose monophosphate pathway exclusive to *Saccharomyces* or the methylotrophic use of WLP have not been reported in the context of PHA synthesis). Alternatively, reduced 1C substrates can be oxidized to CO_2_ and utilized by autotrophic pathways. Figure 6 provides an overview of the methylotrophic pathways used for PHA production.

#### 6.2.1. Methane (CH_4_)

CH_4_ is a major component of natural gas, is present in coal beds, and forms methane clathrates in frozen soils under high pressure. Besides geological processes, CH_4_ is produced by methanogenic archaea, which is widely used for biogas production (containing 50–70% CH_4_). Anaerobic digestion is performed by microbial consortium, where complex biopolymers are mainly degraded to H_2_, CO_2_, and acetate, which are utilized by hydrogenotrophic and acetoclastic methanogens. Biogas can also be further purified to biomethane.

Reported PHA-producing methanotrophs are aerobic. CH_4_ is first oxidized to methanol and then to formaldehyde (HCHO). Based on the subsequent fate of HCHO, methanogens are divided into three groups: Type I utilizes the RuMP cycle, Type II utilizes the serine cycle, and Type X utilizes the RuMP cycle and the Calvin–Benson cycle (reviewed by [91]).

*Methylocystis* sp. GB 25 accumulates 51% PHB in the stationary phase when grown on CH_4_ and when it is limited by nitrogen availability [92]. A mixed culture dominated mainly by *Methylophilus* and *Methylocystis* was capable of achieving 59% PHB accumulation when limited by nitrogen in the stationary phase [93]. *Methylocystis hirsuta* grown using mixotrophic cultivation with natural gas supplied with 0.5% methanol and ethanol in the medium was capable of achieving 73% PHB accumulation [94]. *Methylobacterium organophilum* is able to accumulate 60% PHA with 5% PHV on CH_4_ as its sole carbon source [95]. Other methods for copolymer production require mixotrophic cultivation. The introduction of citrate (250 mg/L) yielded 88% PHA with 55:35:10 PHB:PHV:PHO, and the introduction of propionate (250 mg/L) yielded 60% PHA with 75:25:0 ratio with the same strain [95]. Using an artificial biogas mixture, *Methylocystis hirsuta* accumulated 54% PHA with 25% PHV when supplied with valeric acid (136 mg/L) [96]. An enriched anaerobic sludge accumulated 52% PHA with 33% PHV when valeric acid (50 mg/L) was added [97]. *Methylocystis parvus* accumulated 50% PHA with 10% poly-4-hydroxybutyrate when grown on 1.2 mM 4-hydroxybutyrate [98]. *Methylocystis* sp. WRRC1 accumulated 60% PHA with 50% PHV on 0.34% valerate [99]. It should be noted that CH_4_ can form explosive mixtures with air in the event of an accident, so careful handling is required. CH_4_ is also a greenhouse gas.

#### 6.2.2. Methanol (CH_3_OH)

Hydrogen is often mentioned in the context of a sustainable future, but it suffers from low energy density, problematic storage, and the need to build costly infrastructure. Even with the elimination of fossil fuels, many materials and chemicals still require carbon precursors. Nobel laureate George Andrew Olah proposed the so-called “methanol economy” [100], in which methanol serves as such precursor. Methanol can be synthesized from fossil fuels (syngas), but also from CO_2_, either by chemical hydrogenation or electrocatalytically with H_2_O (current methods and production challenges are reviewed in [101]). Today, methanol is used for the industrial production of many compounds and can be used as a combustible fuel or in fuel cells. It is a liquid at room temperature, has a 3.4 times higher volumetric energy density (MJ/L) than H_2_ compressed at 70 MPa, and a 1.8 times higher volumetric energy density than liquid H_2_ (−253 °C) (see Appendix A for calculations). Therefore, methanol may be a suitable medium for PHA production in the future.

To date, the highest PHA content reported for methanol is for *Methylobacterium extorquens*, which was able to be used to accumulate 53% PHB almost three decades ago [102]. A genetically engineered strain of *Methylobacterium extorquens* AM1 accumulates 44% PHA with 3% PHV content when limited by Co^2+^ concentration, due to the accumulation of propionyl-CoA [103]. However, due to the strong inhibitory effect on cells, the methanol concentration must be kept low (about 0.1%).

#### 6.2.3. Formate (HCOO^−^)

Formic acid is currently produced from fossil fuels; however, similar to methanol, it can also be produced electrochemically or via the direct hydrogenation of CO_2_. Although the volumetric energy density of formic acid is 2.7 times lower than that of MeOH, it is still 1.3 times higher than that of pressurized H_2_ (70 MPa) (see Appendix A for calculations), making it another potential medium.

The aforementioned *Methylobacterium extorquens* is able to accumulate 43% PHB when grown on formate [104]. Formate can also be produced in situ for subsequent microbial consumption (from a more industrial point of view, such system facilitates autotrophy, as CO_2_ is used as the carbon source). Formate can be electrochemically synthesized from CO_2_ and H_2_O separately and then dropped into a bioreactor with *C. necator*, resulting in 34% PHB accumulation [105]. Formate can also be synthesized directly in the cell medium using immobilized formate dehydrogenase on a carbon cloth electrode, NADH, and neutral red dye. *C. necator* with transgenic RuBisCO was able to be used to obtain 485 mg PHB/L (*w*/*w* not determined) in such a system [106].

### 6.3. Non-Methylotrophic Heterotrophy

Most PHA-producing heterotrophs are chemoorganotrophs that oxidize carbon sources for energy, which naturally results in the loss of some carbon source as CO_2_. PHA production has also been reported for photoheterotrophs, which (in theory) could more efficiently utilize the carbon source to produce PHAs, given sufficient illumination.

#### 6.3.1. Food Products

Traditional PHA production uses food products such as sugars and oils as carbon substrates. The use of these substrates can achieve very high yields of PHAs, but the disadvantage is the overlap/competition with food production. The use of extremophilic bacteria can help prevent contamination via PHA non-producing strains and can also result in high PHA content. The halophilic bacterium *Halomonas halophila* is capable of accumulating up to 85% PHB when grown on glucose [107]. A mutant strain of *Halomonas bluephagenesis* can accumulate PHB up to 94% when limited by respiration (knockout of electron transport flavoproteins) on a mixture of glucose (30 g/L) and acetate (3 g/L) [56]. *Halomonas* sp. YLGW01 was able to accumulate 95% PHB during non-sterile cultivation on fructose [108]. However, most publications are related to non-extremophilic bacteria. To reduce costs, refined sugars can be replaced by more crude ones. A bacterial consortium of *Bacillus subtilis* and *C. necator* 5119 grown on cane sugar (90% sucrose) can accumulate 75% PHA [109]. *C. necator* can accumulate up to 92% PHB when grown on maltose-rich brewery wastewater as long as a high C/N ratio is maintained [110]. Using hydrolyzed sugarcane molasse, *C. necator* can accumulate PHB up to 58% [111]. A modified strain of *C. necator* is able to utilize crude corn (maize) starch to produce 63% PHB [65]. The use of oils for PHA production has recently been reviewed by [112]. Palm oil, despite its controversial production, can also be used as a source, and *C. necator* B-10646 can accumulate up to 72% PHA [113]. A modified strain of *C. necator* has also been used to utilize date seed oil with 81% PHA accumulation [114]. As for copolymer production, *C. necator* using fructose and rapeseed oil produced 86% PHA with 17% PHH [115]. *Methylobacterium organophilum* can produce 48% PHA with a ratio of 35:53:12 (PHB:PHV:PHO) on sodium citrate [95].

It should be emphasized that the use of such food industry products can be controversial in the eyes of the public, as it can be seen as a “waste” of food or of arable land (related to the food vs. fuel dilemma). It is also highly dependent on feasible agricultural conditions, intensive use of fertilizers, and it is very water intensive.

#### 6.3.2. Food and Other Industry Waste

An alternative approach is to use food industry wastes such as desugarized sugar beet molasse (the remaining fraction after sugar and betaine isolation from sugar beet). *Bacillus megaterium* yielded 68% PHB content when used as a carbon source [116]. More crude sources require a hydrolysis step, such as inedible rice (*C. necator*, 69% PHA) [117]) or sugarcane bagasse (*Lysinibacillus* sp., 62% PHA) [118]). Another source can be xylose extracted from lignocellulosic biomass. *Bacillus* sp. SM01 yielded 62% PHB on this substrate [119]. It is also worth mentioning a bacterial consortium that utilizes xylose and glucose to produce 1.30 g of PHA per liter. However, the %PHA was not determined [69]. Glycerol is produced in large quantities as a by-product of the transesterification of natural triacylglycerides. *Paracoccus denitrificans* was able to accumulate PHB up to 72% in the stationary growth phase when nitrogen was depleted [120]. Activated sludge mainly containing *Alphaproteobacteria* and *Betaproteobacteria* contained 80% PHA when grown on crude glycerol [121]. Using a mixture of glycerol (20 g/L) and levulinic acid (2 g/L), *C. necator* was able to accumulate 80% PHA when limited by nitrogen [122]. Residues from a specific food industry can also be used, as *Bacillus subtilis* achieved 89% PHA accumulation when grown on onion peels, and *Bacillus siamensis* yielded 82% PHA when grown on orange peels [123]. Whey is a by-product of cheese production and is often discarded as waste. After cheese whey was ultrafiltered and used as a carbon source, *Bacillus megaterium* was able to accumulate 76% PHB [124]. Fats extracted from slaughterhouse waste can also be used for PHA production; a modified *C. necator* strain with high lipase activity was able to accumulate 66% PHA [66]). *C. necator* also accumulated 73% PHA when grown on waste fish oil [125]. Waste cooking oil is also being investigated for PHA synthesis. *Bacillus thermoamylovorans* was able to accumulate 88% PHA [126]. As for copolymer production, sludge palm oil, a solid by-product of palm oil processing, was emulsified with Tween 80 and used as a carbon substrate for *C. necator*, yielding 74% PHA with 22% PHH [127]. Waste rapeseed oil with 1% propanol was also used for *C. necator* to yield 80% PHA with 9% HV [128]. *Halomonas hydrothermalis* was grown on waste frying oil, yielding 69% PHA with 50% PHV when valeric acid (2 g/L) was added and yielding 70% PHA with 8% PHV when propanol (2 g/L) was used [129].

#### 6.3.3. Hydrocarbons

Hydrocarbons are not a typical carbon source for PHA production because petrochemical plastics are cheaper to produce and because their use as a cellular carbon source does not eliminate our dependence on fossil fuels. However, PHAs have been reported to be produced from both aliphatic and aromatic compounds. A mutant strain of *Alcanivorax borkumensis* SK2, a petroleum-degrading marine bacterium, was able to accumulate PHA when fed octadecane at 2.56 g/L (OD_600_ = 1, dry cell mass not determined) [48]. *Pseudomonas putida* CA-3 was able to accumulate up to 42% PHA when grown on styrene with a nitrogen limitation [130]. However, no recent research has been reported on the use of crude hydrocarbons for PHA production.

#### 6.3.4. Short-Chain Carboxylic Acids

Short-chain carboxylic acids, such as acetate and butyrate, are being investigated for PHA production because their metabolic pathways to PHA are relatively short. Most acetic acid today is produced via the carbonylation of methanol with CO, and a small amount is produced via fermentation. Although methanol could be produced via the capture of CO_2_ in the future, the need for CO complicates the process. Recently, the use of acetogens (bacteria and archaea that use WLP) for acetic acid production from CO_2_ and H_2_ has gained great interest. Their metabolism, which produces a large amount of waste compared to biomass (as mentioned in the Section 6.1), would be advantageous here (reviewed in [131]). As for the current PHA production on acetate, *Cobetia* sp. MC34 was able to accumulate 72% PHB in the stationary growth phase [132]. *C. necator* grown on acetate accumulates 72% PHB when limited by nitrogen [133]. Using a mixed carbon source of acetate (16 g/L) and butyrate (8 g/L), the halophilic bacterium *Salinivibrio* sp. TGB19 was able to accumulate PHA up to 89% [134].

Similar to acetic acid, butyric acid is mostly produced using syngas and propene to form butyraldehyde, which is subsequently oxidized to butyric acid, but it can also be produced via fermentation. An enriched culture dominated by *Plasticicumulans acidivorans* was capable of obtaining 88% PHB production, both on butyrate alone and on a mixture of acetate with butyrate. Butyrate was the preferred carbon source for the bacteria [135]. *C. necator* is able to accumulate 66% PHA with up to 13% PHV when using butyrate as its sole carbon source [136]. The cultivation of *C. necator* on butyrate supplied by γ-valerolactone resulted in 78% PHA accumulation with 31% PHV [137].

Propionate is often used to promote the synthesis of PHA copolymers. Most of it is produced industrially from fossil fuels via the carbonylation of ethylene, but it can also be produced via fermentation. *Methylobacterium organophilum* can accumulate 37% PHA with a PHB:PHV:PHO ratio of 37:56:7 when grown on propionate as its sole carbon source [95].

#### 6.3.5. Photo-Organoheterotrophy

Some photo-organoheterotrophs are capable of PHA accumulation. *Dinoroseobacter* sp. JL1447 accumulated 72% PHA when grown on sodium acetate at a high C/N ratio. The use of a light/dark cycle resulted in a quarter increase in PHA content compared to the unilluminated strain [138]. *Oscillatoria okeni* was able to produce 14% PHA at 6% PHV using only CO_2_ during nitrogen-limited conditions (as mentioned in the Section 6.1); however, when supplied with 0.4% acetate, PHA increased to 42% at 7% PHV [78]. The introduction of acetate resulted in 81% PHB content when the autotrophically grown mutant *Synechocystis* sp. PCC 6803 was starved for 20 days in a phosphorus- and nitrogen-free medium [139]. A consortium of phototrophic bacteria grown on butyrate (3 g/L) and acetate (1.75 g/L) was able to accumulate up to 67% PHA when limited by nitrogen, with an undetermined amount of PHV [140]. *Rhodobacter sphaeroides* can accumulate up to 72% PHA with 2% PHV when grown on acetate supplemented with malate under nitrogen-limited conditions [141].

## 7. Discussion and Outlook

PHAs have the potential to replace petrochemical plastics due to their biological production and biodegradability. For sustainable PHA production, in addition to using safe solvents for extraction, the carbon source should be independent of fossil fuels. There are several possibilities for large-scale PHA production in the future based on the carbon sources available.

**Figure 7:**
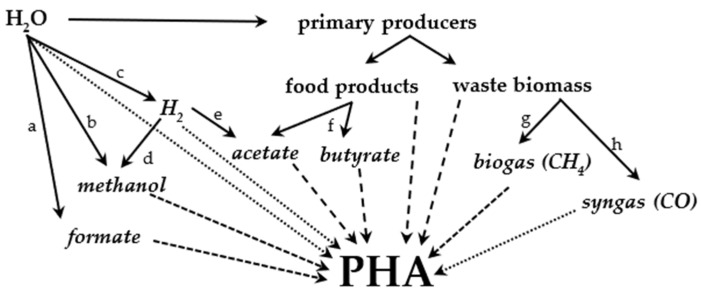
Overview of PHA production pathways. Terms in italics refer to sources that are or can be currently mass-produced using fossil fuels. Dotted lines: autotrophs. Densely dashed lines: methylotrophs. Loosely dashed lines: other heterotrophs. Solid lines: processes related to PHA production: a—electrochemical synthesis of formate, b—electrochemical synthesis of methanol, c—production of hydrogen from water, d—production of methanol by hydrogenation, e—production of acetate by acetogens, f—fermentation of sugars, g—anaerobic digestion, h—biomass gasification.

Food products can be used for PHA production with high yields. However, such production requires feasible agricultural conditions that are not easily met worldwide and could be perceived negatively by the public. Worldwide PHA production using more ubiquitous carbon sources could be an alternative. Lignocellulose is an abundant and globally available carbon source that can be used either directly or via the conversion to CH_4_-rich biogas for use by methylotrophs. Sustainably produced methanol (or formate) can also become a ubiquitous carbon source for methylotrophs, but such use is highly dependent on its future price (the production of carbon-neutral fuels has recently been reviewed by [142]). Direct use of CO_2_ by autotrophs is also feasible. Sufficient sunlight is available on most of the Earth (and could be augmented by low-energy electric lights if needed). Another source of reducing energy for autotrophs is H_2_, which is often mentioned in connection with a sustainable future. If the price of sustainably produced H_2_ decreases sufficiently, PHA production with hydrogenotrophs would not be limited to any location. The aerobic hydrogenotrophic production of copolymers with mcl-PHAs is also very feasible. Anaerobic hydrogenotrophs can also be used for acetate production (not requiring food), which can be used as a reduced carbon source for (photo-)heterotrophic PHA-producing strains. Figure 7 provides an overview of the pathways leading to PHA production discussed in this article. Table 4 lists all the strains discussed in this article.

## Figures and Tables

**Table 1:** Comparison of mechanistic properties of various PHA copolymers.

PHB:PHV:PHH	T_m_ (°C)	TS (MPa)	EaB (%)	Reference
93:0:17	120	20	850	[6]
85:0:15	115	23	760	[6]
69:24:7	129, 139	20	710	[7]
90:0:10	127	21	400	[6]
71:26:3	131, 143	12	324	[8]
80:16:4	140, 151	12	321	[8]
91:2:7	144	22	312	[8]
87:9:4	142, 153	21	287	[8]
66:32:2	91, 148	5	282	[7]
82:15:3	147	6	84	[7]
94:3:3	129, 144	4	79	[7]
14:85:1	89	14.5	78	[7]
80:20:0	170–175 *	30.8	54	[9]
0:100:0	130–135 *	31.2	14	[9]
P4HB ^1^	53	104	1000	[10]
PHP ^2^	78	28.3	634	[11]
PP ^3^	170	34	400	[12]

T_m_—melting temperature, TS—tensile strength, EaB—elongation at break, PHB—poly(3-hydroxybutyrate), PHV—poly(3-hydroxyvalerate), PHH—poly(hydroxyhexanoate), *—stated as molding temperature, 1—poly(4-hydroxybutyrate), 2—polyhydroxypropionate, 3—polypropylene (reference as a petrochemical plastic).

**Table 2:** Overview of types of “-trophs”.

Source of Energy	Source of Electrons	Source of Carbon	
*chemical bond*	*inorganic compound*	*oxidized C*	
**chemo-**	**-litho-**	**-auto-**	**-troph**
**photo-**	**-organo-**	**-hetero-**	
*electromagnetic radiation*	*organic compound*	*reduced C*	

**Table 3:** Overview of metabolism of PHA producers discussed in this article.

Autotrophy	Methylotrophy	Heterotrophy
CO_2_ + sunlight	Methane	Food products
CO_2_ + H_2_	Methanol	Food and other industry waste
CO	Formate	Hydrocarbons
		Short carboxylic acids
		Carboxylic acids + sunlight

**Table 4:** Overview of strains discussed in this article.

Strain	Growth Source	PHA	PHB	PHV	Other	Reference
*Synechocystis* sp. PCC 6714	CO_2_ + H_2_O + γ		37			[76]
*Synechocystis* sp. PCC 6803	CO_2_ + H_2_O + γ		39			[77]
*Oscillatoria okeni*	CO_2_ + H_2_O + γ	14		6		[78]
*Oscillatoria okeni*	CO_2_ + H_2_O + γ + acetate	42		7		[78]
*Anabena* sp.	CO_2_ + H_2_O + γ	40		5		[79]
*Anabena* sp.	CO_2_ + H_2_O + γ + acetate	14		24		[79]
*C. necator*	CO_2_ + H_2_		85			[80]
*C. necator*	CO_2_ + H_2_	55			20 (PHH)	[81]
*C. necator*	CO_2_ + H_2_	46			39 (PHD)	[81]
*C. necator*	CO_2_ + H_2_	42			55 (PHO)	[81]
*Seliberia carboxydohydrogena*	CO	63				[87]
*Clostridrium autoethanogenum*	CO		6			[89]
*Methylocystis sp. GB 25*	CH_4_		51			[92]
*Methylophilus + Methylocystis*	CH_4_		59			[93]
*Methylocystis hirsuta*	CH_4_ + methanol, ethanol		73			[94]
*Methylobacterium organophilum*	CH_4_	60		5		[95]
*Methylobacterium organophilum*	CH_4_ + citrate	88		55	35 (PHO), 10 (PHD)	[95]
*Methylobacterium organophilum*	CH_4_ + propionate	60		75	25 (PHO)	[95]
*Methylocystis hirsuta*	CH_4_ + valerate	54		25		[96]
Enriched sludge	CH_4_ + valerate	52		33		[97]
*Methylocystis parvus*	CH_4_ +4-hydroxybutyrate	50			10 (P4HB)	[98]
*Methylocystis* sp. WRRC1	CH_4_ + valerate	60		50		[99]
*Methylobacterium extorquens*	methanol		53			[102]
*Methylobacterium extorquens*	methanol		44			[103]
*Methylobacterium extorquens*	formate		43			[104]
*C. necator*	Formate-dropping		34			[105]
*Halomonas halophila* *Halomonas bluephagenesis*	glucose		85			[107]
	glucose + acetate		94			[56]
*Halomonas sp. YLGW01*	fructose		95			[108]
*Bacillus subtilis + C. necator*	sugarcane sugar	75				[109]
*C. necator*	brewery wastewater		92			[110]
*C. necator*	hydrolyzed sugarcane molasse		58			[111]
*C. necator*	crude corn starch		63			[65]
*C. necator*	palm oil	72				[112]
*C. necator*	date seed oil	81				[114]
*C. necator*	fructose + rapeseed oil	86			17 (PHH)	[115]
*Methylobacterium organophilum*	citrate	48	35	53	12 (PHO)	[95]
*Bacillus megatherium*	desugarized sugar beet molasse		68			[116]
*C. necator*	hydrolyzed inedible rice	69				[117]
*Lysinibacillus* sp.	hydrolyzed sugarcane bagasse	62				[118]
*Bacillus* sp. SM01	xylose form lignocellulose	62				[119]
*Paracoccus denitrificans*	glycerol		72			[120]
Activated sludge	glycerol	80				[121]
*C. necator*	glycerol + levulinic acid	80				[122]
*Bacillus subtilis*	onion peels	89				[123]
*Bacillus siamensis*	orange peels	82				[123]
*Bacillus megatherium*	cheese whey		76			[124]
*C. necator*	slaughterhouse waste fats	66				[66]
*C. necator*	waste fish oil	73				[125]
*Bacillus thermoamylovorans*	waste cooking oil	88				[126]
*C. necator*	sludge palm oil	74			22 (PHO)	[127]
*C. necator*	waste rapeseed oil + propanol	80		9		[125]
*Halomonas hydrothermalis*	waste frying oil + valeric acid	69				[129]
*Halomonas hydrothermalis*	waste frying oil + propanol	70				[129]
*Pseudomonas putida*	styrene	42				[130]
*Cobetia* sp. MC34	acetate		72			[132]
*C. necator*	acetate		72			[133]
*Salinivibrio* sp. TGB19	acetate + butyrate	89				[134]
*Plasticicumulans acidovorans*	butyrate		88			[135]
*C. necator*	butyrate	66		13		[136]
*C. necator*	butyrate + 4-valerolactone	78		31		[137]
*Methylobacterium organophilum*	propionate	37	37	56	7 (PHD)	[95]
*Dinoroseobacter* sp. JL1447	acetate + γ	72				[138]
*Oscillatoria okeni*	acetate + γ	42	7			[78]
*Synechocystic* sp. PCC 6803	acetate + γ	81				[139]
Bacterial consortium	butyrate + acetate + γ	67				[140]
*Rhodobacter sphaeroides*	acetate + malate + γ	72	2			[141]

Numbers refer to % in dry cell mass. When more types of polymers are present, values refer to % in total PHA, not in dry cell mass. When only PHB is reported, it can be considered as total PHA detected. PHH—polyhydroxyhexanoate, PHO—polyhydroxyoctanoate, PHD—polyhydroxydodecanoate, P4HB—poly-4-hydroxybutyrate, γ—electromagnetic radiation (sunlight).

## Data Availability

No new data were created or analyzed in this study. Data sharing is not applicable to this article.

## Appendix A

Table A1 shows volumetric energy densities (EVs) of certain fuels mentioned in this paper in relation to the growth sources for bacterial strains. These values were calculated as the enthalpy of combustion (ΔH^°^_comb_, “higher heating value”) related to a volume of 1 L of fuel. Standard formation entropy (Δ_f_H^°^_comb_) values were taken from the NIST database of the National Institute of Standards and Technology [143]; densities and molecular weights were taken from the PubChem database of the National Library of Medicine [144]. The value for 70 MPa H_2_ was taken from [145]. Only values relevant for calculations are shown.

**Table A1:** Overview of tabulated values used for calculations.

Compound	Δ_f_H^°^_comb_ (kJ/mol)	ρ (g/cm^3^)	M (g/mol)
MeOH (l)	−238.4	0.792	32.042
HCOOH (l)	−425.09	1.22	46.025
CO_2_ (g)	−393.51		
H_2_O (l)	−285.83		
H_2_ (l, −253 °C)		0.071	2.016
H_2_ (g, 70 MPa)		37	2.016

References: Δ_f_H^°^_comb_: [143], ρ: [144], ρ (H_2_, g, 70 MPa): [145], M: [144].

Hydrogen: H_2_ + ^1^/_2_ O_2_ → H_2_O

ΔH^°^_comb_ = −285.83 kJ/mol

Liquid H_2_:

c = 35.2 mol/L

EV = −10.06 MJ/L

Gaseous H_2_:

c = 18.35 mol/L

EV = −5.25 MJ/L

Formic acid: HCOOH + ^1^/_2_ O_2_ → CO_2_ + H_2_O

ΔH^°^_comb_ = −393.51 + (−285.83) − (−425.09) = −254.25 kJ/mol

c = 26.51 mol/L

EV = −6.74 MJ/L

Methanol: CH_3_OH + O_2_ → CO_2_ + 2 H_2_O

ΔH^°^_comb_ = −393.51 + (−285.83 × 2) − (−238.4) = −726.77 kJ/mol

c = 24.7 mol/L

EV = −17.95 MJ/L
